# Safety and tolerability of sitagliptin in clinical studies: a pooled analysis of data from 10,246 patients with type 2 diabetes

**DOI:** 10.1186/1472-6823-10-7

**Published:** 2010-04-22

**Authors:** Debora Williams-Herman, Samuel S Engel, Elizabeth Round, Jeremy Johnson, Gregory T Golm, Hua Guo, Bret J Musser, Michael J Davies, Keith D Kaufman, Barry J Goldstein

**Affiliations:** 1 Merck Research Laboratories, Rahway, NJ USA

## Abstract

**Background:**

In a previous pooled analysis of 12 double-blind clinical studies that included data on 6,139 patients with type 2 diabetes, treatment with sitagliptin, a dipeptidyl peptidase-4 (DPP-4) inhibitor, was shown to be generally well tolerated compared with treatment with control agents. As clinical development of sitagliptin continues, additional studies have been completed, and more patients have been exposed to sitagliptin. The purpose of the present analysis is to update the safety and tolerability assessment of sitagliptin by pooling data from 19 double-blind clinical studies.

**Methods:**

The present analysis included data from 10,246 patients with type 2 diabetes who received either sitagliptin 100 mg/day (N = 5,429; sitagliptin group) or a comparator agent (placebo or an active comparator) (N = 4,817; non-exposed group). The 19 studies from which this pooled population was drawn represent the double-blind, randomized studies that included patients treated with the usual clinical dose of sitagliptin (100 mg/day) for between 12 weeks and 2 years and for which results were available as of July 2009. These 19 studies assessed sitagliptin taken as monotherapy, initial combination therapy with metformin or pioglitazone, or as add-on combination therapy with other antihyperglycemic agents (metformin, pioglitazone, a sulfonylurea ± metformin, insulin ± metformin, or rosiglitazone + metformin). Patients in the non-exposed group were taking placebo, metformin, pioglitazone, a sulfonylurea ± metformin, insulin ± metformin, or rosiglitazone + metformin. The analysis used patient-level data from each study to evaluate between-group differences in the exposure-adjusted incidence rates of adverse events.

**Results:**

Summary measures of overall adverse events were similar in the sitagliptin and non-exposed groups, except for an increased incidence of drug-related adverse events in the non-exposed group. Incidence rates of specific adverse events were also generally similar between the two groups, except for increased incidence rates of hypoglycemia, related to the greater use of a sulfonylurea, and diarrhea, related to the greater use of metformin, in the non-exposed group and constipation in the sitagliptin group. Treatment with sitagliptin was not associated with an increased risk of major adverse cardiovascular events.

**Conclusions:**

In this updated pooled safety analysis of data from 10,246 patients with type 2 diabetes, sitagliptin 100 mg/day was generally well tolerated in clinical trials of up to 2 years in duration.

## Background

The safety and tolerability of sitagliptin, a dipeptidyl peptidase-4 (DPP-4) inhibitor, were found to be generally comparable to non-sitagliptin treatments in a pooled analysis of 12 double-blind, randomized, controlled studies comprising data on 6,139 patients with type 2 diabetes that was published in 2008 [1]. Concurrent with the increased use of sitagliptin (administered as either sitagliptin tablets or as sitagliptin/metformin fixed-dose combination tablets) in clinical practice, additional clinical studies have expanded the controlled trial dataset that informs the safety experience with sitagliptin. In this updated report, the safety and tolerability of sitagliptin 100 mg/day were examined in a pooled analysis of patient-level data from 19 double-blind, randomized studies that included 10,246 patients with type 2 diabetes. Endpoints of particular interest regarding the safety of medications used in patients with type 2 diabetes (e.g., cardiovascular adverse events [2] and adverse events of malignancy [3]) were evaluated in greater detail, as were adverse events potentially related to the mechanism of action of specific antihyperglycemic agents (AHAs) (e.g., hypoglycemia, gastrointestinal intolerance, bone fractures [4-7]), and events postulated to be potentially related to inhibition of the DPP-4 enzyme [8-10].

## Methods

Similar to the previous report that assessed pooled, patient-level data [1], the present analysis evaluated the usual clinical dose of sitagliptin (100 mg/day) approved for use in patients with type 2 diabetes. The pooled population was drawn from all 19 multicenter, U.S. or multinational, double-blind, parallel-group studies conducted by Merck Sharp & Dohme Corp., in which patients were randomized to receive sitagliptin 100 mg/day (or comparator) for at least 12 weeks and up to 2 years (the duration of the longest studies) and for which results were available as of July 2009. Protocols were reviewed and approved by appropriate ethical review committees and authorities for each clinical site; all patients were to have provided written informed consent. These studies assessed sitagliptin when used as monotherapy, initial combination therapy with either metformin or pioglitazone, or add-on combination therapy with other AHAs including metformin, pioglitazone, a sulfonylurea (with and without metformin), insulin (with and without metformin), or metformin + rosiglitazone. Patients not receiving sitagliptin (i.e., the non-exposed group) received placebo, metformin, pioglitazone, a sulfonylurea, (with and without metformin), insulin (with and without metformin), or metformin + rosiglitazone. From each contributing study, the pooling was conducted by including portions of controlled, double-blind studies that had parallel treatment groups with concurrent exposures to sitagliptin 100 mg/day (primarily administered as 100 mg once daily) or other treatments (either placebo or active-comparator; see Table 1 for a listing of studies). Sitagliptin 100 mg/day was administered as 50 mg twice daily, either alone or in combination with metformin, in 4 studies: two dose-range finding studies [11,12], the study of initial combination therapy with sitagliptin and metformin that simulated the twice-daily administration of a fixed-dose combination of sitagliptin and metformin [13], and the study in which the fixed-dose combination tablet of sitagliptin and metformin was administered twice daily [14,15]. Studies conducted only in Japan were excluded from all analyses; a lower starting dose of sitagliptin has been separately developed in Japan. Similarly, the pooling did not include results from patients with renal insufficiency randomized to receive dose-adjusted sitagliptin at less than 100 mg/day, including results from a special populations study of patients with moderate or severe/end-stage renal insufficiency who were randomized to receive either 50 or 25 mg once daily [16].

**Table 1:** Studies and treatment arms included in the primary analysis

Study	Study Design	Sitagliptin 100 mg/day Group (N = 5429)	n	Non-exposed Group (N = 4817)	n	Reference*
**P010: **twice-daily dose-range finding study	106-week active-controlled period	Sitagliptin 50 mg b.i.d. switched to Sitagliptin 100 mg q.d.	122	Glipizide	123	[11]
**P014**: once-daily dose-range finding study	12-week placebo-controlled period and 94-week active- controlled period	-Sitagliptin 100 mg q.d. -Sitagliptin 50 mg b.i.d. switched to Sitagliptin 100 mg q.d.	110 111	Placebo (12 weeks) switched to metformin (94 weeks)	111	[12]
**P019: **placebo-controlled add-on to pioglitazone study	24-week placebo-controlled period	Sitagliptin 100 mg q.d. + pioglitazone	175	Placebo + pioglitazone	178	[48]
**P020: **placebo-controlled add-on to metformin study	24-week placebo-controlled period and 80-week active- controlled period	Sitagliptin 100 mg q.d. + metformin	464	Placebo + metformin (24 weeks) switched to glipizide + metformin (80 weeks)	237	[49]
**P021**: placebo-controlled monotherapy study	24-week placebo-controlled period	Sitagliptin 100 mg q.d.	238	Placebo	253	[50]
**P023: **placebo-controlled monotherapy study	18-week placebo-controlled period and 36-week active- controlled period	Sitagliptin 100 mg q.d.	205	Placebo (18 weeks) switched to pioglitazone (36 weeks)	110	[51]
**P024: **active-comparator controlled add-on to metformin study	104-week active-controlled period	Sitagliptin 100 mg q.d. + metformin	588	Glipizide + metformin	584	[23,24]
**P035: **placebo-controlled add-on to glimepiride, alone or in combination with metformin study	24-week placebo-controlled period and 30-week active- controlled period	Sitagliptin 100 mg q.d. + glimepiride (± metformin)	222	Placebo + glimepiride (± metformin) (24 weeks) switched to pioglitazone + glimepiride (± metformin) (30 weeks)	219	[25]
**P036: **placebo- and active-controlled study of initial combination use of sitagliptin and metformin	24-week placebo-controlled period; 80-week active-controlled period	-Sitagliptin 100 mg q.d. -Sitagliptin 50 mg b.i.d./metformin 500 mg b.i.d. -Sitagliptin 50 mg b.i.d./metformin 1000 mg b.i.d.	179 190 182	-Placebo (24 weeks) switched to metformin (80 weeks) -Metformin 500 mg b.i.d. -Metformin 1000 mg b.i.d.	176 182 182	[13,52,53]
**P040: **placebo-controlled monotherapy study	18-week placebo-controlled period	Sitagliptin 100 mg q.d.	352	Placebo	178	[54]
**P047: **placebo-controlled monotherapy study in elderly patients	24-week placebo-controlled period	Sitagliptin 100 mg q.d	91	Placebo	92	[55]
**P049: **active-comparator controlled monotherapy study	24-week active-controlled period	Sitagliptin 100 mg q.d	528	Metformin	522	[56]
**P051: **placebo-controlled add-on to insulin, alone or in combination with metformin study	24-week placebo-controlled period	Sitagliptin 100 mg q.d + insulin (± metformin)	322	Placebo + insulin ± metformin	319	[26]
**P052: **placebo-controlled add-on to metformin and rosiglitazone study	54-week placebo-controlled period	Sitagliptin 100 mg q.d. + metformin and rosiglitazone	170	Placebo + metformin and rosiglitazone	92	[57]
**P053: **placebo-controlled add-on to metformin study	30-week placebo-controlled period	Sitagliptin 100 mg q.d. + metformin	96	Placebo + metformin	94	[58]
**P061: **placebo- and active-controlled mechanism of action factorial study	12-week placebo-controlled period	-Sitagliptin 100 mg q.d. -Sitagliptin 100 mg q.d. + pioglitazone	52 52	-Pioglitazone -Placebo	54 53	[28]
**P064: **active-comparator controlled study of initial combination use of sitagliptin and pioglitazone	54-week active-controlled period	Sitagliptin 100 mg q.d + pioglitazone	261	Placebo + pioglitazone	259	[29]
**P079: **active-comparator controlled study of initial combination use of sitagliptin/metformin FDC (MK-0431A)	44-week active-controlled period	Sitagliptin 50 mg + metformin 1000 mg b.i.d. (FDC)	625	Metformin 1000 mg b.i.d.	621	[14,15]
**P801: **placebo- and active-controlled add-on to metformin study	18-week placebo-controlled period	Sitagliptin 100 mg q.d.	94	-Rosiglitazone -Placebo	87 91	[59]

*References are for the initial phases of the studies that had extension or continuation phases, unless a reference is provided for the results beyond the initial phase.

q.d. = once daily; b.i.d. = twice daily

Baseline demographics and disease characteristics were summarized for the pooled population and for each treatment group. For race/ethnicity, the data capture system was changed (2008) to reflect the US FDA guidance [17] regarding the collection of race and ethnicity data in clinical trials (i.e., categorization of Hispanic/Latino background as ethnicity, independent of race). Consequently, data reflecting Hispanic/Latino background were not collected in a consistent manner across all studies, and cannot be pooled and summarized.

In each study included in this pooled analysis, investigators were to report adverse events (serious and non-serious) that occurred during the conduct of the study. Further, all serious adverse events occurring within 14 days following the last dose of blinded study drug were to have been reported. This pooled analysis used patient-level data from each study to assess the incidence rates of specific adverse events that occurred following initiation of double-blind study drug. These events were encoded in a uniform manner using the Medical Dictionary for Regulatory Activities (MedDRA version 12.0), in which terms for specific adverse events that are alike or pertain to the same organ system are categorized by System Organ Class (SOC). To account for potential differences between groups in duration of exposure to treatment, reports of adverse events are expressed as exposure-adjusted incidence rates (numbers of patients with events per 100 patient-years). These analyses were based upon the time to the first (incident) event, calculated as follows: incident event rate = 100 * (total number of patients with ≥ 1 event during eligible exposure period per total patient-years of exposure). The incident event rate per 100 patient-years is referred to as the "incidence rate" throughout the manuscript. For patients for whom an event was reported, the patient-years of exposure were calculated as the time from the first dose of sitagliptin (or comparator) at randomization to the time that the first post-randomization event occurred. For patients without an event, the patient-years of exposure were calculated as the time from the first dose to 14 days after the last dose of study medication (i.e., sitagliptin or comparator).

Most of the studies in this analysis included open-label glycemic rescue therapy. Glycemic rescue therapies included metformin, a thiazolidinedione, a sulfonylurea, or an insulin dose increase (in the add-on to insulin study), and were to have been initiated based upon progressively stricter, protocol-specified hyperglycemic criteria. When initiated, glycemic rescue therapy was added to the ongoing, blinded study medication to which patients had been randomized. The primary analyses in this pooled population present results of post-randomization events reported to have occurred during a given study, including those events with onset after the initiation of glycemic rescue therapy, unless otherwise specified. Results were summarized by SOC for specific adverse events occurring with a reported incidence rate of at least 1 incident event per 100 patient-years for either the sitagliptin-exposed or the non-exposed group. Incidence rates of specific adverse events reported with like terms (e.g., "urinary tract infection", and "urinary tract infection, bacterial") were pooled and assessed as sensitivity analyses in some instances. Since drug-related and serious clinical adverse events are a subset of all adverse events and reported less frequently, these events were summarized using a lower cut point (i.e., an incidence rate of at least 0.2 incident events per 100 patient-years). Differences between treatment groups and the associated 95% confidence intervals (CI) were calculated using the Miettinen and Nurminen method, stratified by study [18]. No statistical adjustments were performed for multiple comparisons. All analyses were performed using SAS Version 9.1.

Although both analyses used patient-level data and assessed all reported adverse events, there were some differences in the methodologies used in the present analysis relative to the previously published pooled analysis of the safety of sitagliptin [1]. The primary approach in the present analysis evaluated exposure-adjusted incidence rates that included data after initiation of glycemic rescue therapy. In contrast, the previous analysis examined crude (i.e., not exposure-adjusted) incidence rates and excluded data after initiation of glycemic rescue therapy. In addition, the present analysis employed stratification by study to account for potential differences among studies (such as sample size or randomization ratios). To assess the robustness of the conclusions from the primary analysis, the results were also analyzed after excluding data following initiation of glycemic rescue therapy. In addition, crude incidence rates were compared between groups. Results from these analyses were generally consistent with the primary analyses and are not presented herein.

### Adverse Events of Interest

For most studies, prespecified adverse events of interest included hypoglycemia and predefined gastrointestinal (GI) adverse events (including diarrhea, nausea, vomiting and a composite abdominal pain term, which included abdominal pain, upper and lower abdominal pain, and abdominal and epigastric discomfort). For all of the trials that were pooled for this analysis, hypoglycemia was based upon investigator interpretation of clinical symptoms, without the requirement for a concurrent glucose determination. In contrast to the general analysis of adverse events, analyses of hypoglycemia and predefined GI adverse events excluded data following initiation of glycemic rescue therapy to avoid the confounding influence of medications that could cause hypoglycemia or gastrointestinal symptoms. In addition, a separate pooled analysis was performed, using studies and portions of studies that did not include a sulfonylurea or insulin, to characterize the rate of hypoglycemia with sitagliptin relative to comparators not generally associated with an increased risk for hypoglycemia (i.e., metformin and pioglitazone, as well as placebo). For the predefined GI adverse events of interest, a separate pooled analysis was conducted, excluding studies and portions of studies in which patients initiated metformin, to characterize the rate of these GI events with sitagliptin relative to comparators generally not associated with an increased risk for GI events. The analysis of reports of pancreatitis, an additional gastrointestinal adverse event of recent interest, is presented elsewhere [19].

#### Cardiovascular

An analysis using major adverse cardiovascular events (MACE) that focused on ischemic events (see Appendix I for list of terms) and cardiovascular deaths was performed to inform on the cardiovascular safety of sitagliptin in patients with type 2 diabetes. There was no formal adjudication of these cardiovascular adverse events. Exposure-adjusted risk ratios were calculated using the exact procedures for the Poisson processes [20]. A sensitivity analysis was conducted using the Mantel-Haenszel approach, a method which can accommodate studies with no events [21]. Both analyses were stratified by study.

#### Malignancy

The Neoplasms SOC contains all adverse event terms for malignancies, as well as terms for a variety of non-malignant neoplasms. The events within this SOC were reviewed and categorized as malignancies or residual (i.e., non-malignant) neoplasms. Incidence rates and between-group differences were computed for individual and combined events (i.e., all malignancies, all residual events).

#### Bone Fracture

Analyses of the adverse event of bone fracture used a composite endpoint of all identified fractures in the database reported as either specific adverse events or results of a radiologic evaluation, or those described within a narrative of a serious adverse event. The primary analysis was performed excluding data following initiation of glycemic rescue therapy to avoid the potentially confounding influence of glycemic rescue therapy, since pioglitazone was used as rescue therapy in a number of studies. To improve sensitivity, the primary analysis also excluded face or skull fractures and those related to high-impact trauma (e.g., motor vehicle accident or fall from greater than 5 feet). An additional analysis evaluated the composite endpoint of all bone fractures regardless of location or association with high-impact trauma. To control for the confounding effects of initiating treatment with a thiazolidinedione as part of blinded study therapy, a separate pooled analysis was performed that excluded data from studies and portions of studies in which a thiazolidinedione (i.e., pioglitazone or rosiglitazone) was initiated as part of blinded study therapy in any treatment group.

#### Infection

Due to theoretical concerns regarding the potential role of DPP-4 in immune function, overall and specific adverse events of infection were examined to identify potential infections or trends suggestive of compromised immunity.

#### Angioedema

Angioedema events and angioedema-related events, based upon an expanded version of the Standard MedDRA Query (SMQ) that included anaphylactic reactions and hypersensitivity (Appendix II), were summarized by treatment group for the periods with and without exposure to an angiotensin-converting enzyme (ACE) inhibitor. Exposure to an ACE inhibitor was defined as the total days of use of an ACE inhibitor during the double-blind treatment period, with patients contributing to patient-years of exposure to an ACE inhibitor for the actual period of time that they were reported to have been taking an ACE inhibitor and to patient-years of non-exposure for the actual period of time that they were reported not to have been taking an ACE inhibitor.

#### Liver Function Tests

Percentages of patients meeting predefined limits of change (PDLC) for alanine aminotransferase (ALT) and aspartate aminotransferase (AST) were compared between groups using the previously described statistical analysis.

## Results

### Patient Characteristics and Exposure

In this pooled analysis, there were 10,246 patients overall, with 5,429 in the sitagliptin group and 4,817 in the non-exposed group. At baseline, patients in the total cohort had an average age of 55 years (range: 19 to 91 years; 18% ≥ 65 years), a median duration of diabetes of 3.5 years, and a mean HbA_1c _of 8.4%. Men comprised 54% of this cohort. The participating population was 64% White, 15% Asian, and 6% Black. The per study contribution from Hispanic patients varied from 0% to 36% depending on the method of data capture (see Methods) as well as the study population. At baseline, 11% of patients had a history of cardiovascular disease, and 82% had additional cardiovascular risk factors besides type 2 diabetes and cardiovascular disease, including hypertension (55%), history of dyslipidemia/hypercholesterolemia (50%), and history of smoking (40%). There were no meaningful differences between treatment groups in these baseline characteristics or in the frequency or type of baseline medical conditions or other medications used.

The mean exposure to study drug and total patient-years in study were greater in the sitagliptin group relative to the non-exposed group: 282 dosing days (range = 1 to 791) relative to 259 dosing days (1 to 801), respectively. The cumulative patient exposure was 4,709 patient-years for the sitagliptin group and 3,943 patient-years for the non-exposed group. In the sitagliptin group, 1,805 patients were treated for at least 1 year, with 584 of these patients treated for 2 years; the corresponding numbers of patients were 1,320 and 470 in the non-exposed group. In this pooled analysis of studies 12 weeks to 2 years in duration, the proportions of patients discontinuing treatment were 33% in the sitagliptin group and 35% in the non-exposed group, with generally similar reasons for discontinuations in the two treatment groups (Table 2).

**Table 2:** Overall disposition

	Sitagliptin 100 mg	Non-exposed
RANDOMIZED, N	5429	4817
		
	n (%)	n (%)
DISCONTINUED	1818 (33.5)	1694 (35.2)
Reason for discontinuation		
Adverse event	239 (4.4)	216 (4.5)
Lack of efficacy*	614 (11.3)	520 (10.8)
Lost to follow-up	222 (4.1)	180 (3.7)
Protocol violation	98 (1.8)	99 (2.1)
Protocol-specific criteria	61 (1.1)	55 (1.1)
Withdrawal of consent	336 (6.2)	367 (7.6)
Other reasons^†^	248 (4.6)	257 (5.3)

* Includes patients not meeting the protocol-specified, progressively stricter glycemic rescue criteria and/or not meeting the investigator's expectations of glycemic improvement.

^† ^Includes pregnancy, physician decision, patient moved, site terminated, and other.

### Safety and Tolerability

Summary measures of reported adverse events and deaths were similar in the sitagliptin and non-exposed groups, except that the incidence rate of drug-related adverse events was higher in the non-exposed group (Table 3), primarily due to the greater incidence rate of adverse events of hypoglycemia reported for the non-exposed group. Incidence rates for each SOC are in Table 4. There were 3 SOCs (Blood and Lymphatic System Disorders, Skin and Subcutaneous Disorders, and Metabolism and Nutrition Disorders) for which the 95% CI for the between-group difference in incidence rate excluded 0. The between-group difference in incidence rate of pooled adverse events in the Blood and Lymphatic System Disorders SOC was primarily due to slightly higher incidence rates of the specific adverse events of anemia and iron-deficiency anemia in the sitagliptin group. For the specific adverse event of anemia, the incidence rates were 0.4 and 0.2 per 100 patient-years in the sitagliptin and non-exposed groups, respectively, with a between-group difference (95% CI) of 0.2 (-0.1, 0.4); for iron-deficiency anemia, the incidence rates were 0.3 and 0.1, with a between-group difference (95% CI) of 0.2 (-0.0, 0.4). For the Skin and Subcutaneous Disorders SOC, the adverse events of contact dermatitis (0.7 vs. 0.3), macular rash (0.3 vs. 0.1), and acne (0.2 vs. 0.0) accounted for the higher incidence rate in the sitagliptin group relative to the non-exposed group, respectively. The between-group difference in the incidence rate of pooled adverse events in the Metabolism and Nutrition Disorders SOC was primarily due to a higher incidence rate of hypoglycemia in the non-exposed group (discussed below).

**Table 3:** Adverse event summary

	Incidence Rate per 100 Patient-years^†^		
	Sitagliptin 100 mg	Non-exposed	Difference between Sitagliptin and Non-exposed (95% CI)*
With one or more adverse events	153.5	162.6	-7.6 (-15.6, 0.3)
With drug-related^‡ ^adverse events	20.0	26.8	-6.4 (-8.7, -4.1)
With serious adverse events	7.8	7.9	-0.1 (-1.3, 1.1)
With serious drug-related^‡ ^adverse events	0.4	0.3	0.1 (-0.1, 0.4)
Who died	0.3	0.5	-0.2 (-0.5, 0.1)
Discontinued due to adverse events	4.8	5.2	-0.5 (-1.5, 0.4)
Discontinued due to drug-related^‡ ^adverse events	1.7	2.3	-0.5 (-1.1, 0.1)
Discontinued due to serious adverse events	1.7	1.7	-0.0 (-0.6, 0.5)
Discontinued due to serious drug-related^‡ ^adverse events	0.2	0.1	0.1 (-0.1, 0.3)

CI = confidence interval

^†^100 * (number of patients with ≥ 1 event/person years of follow-up time).

* Between-group difference and 95% CI based on stratified analysis. Positive differences indicate that the incidence rate for the sitagliptin group is higher than the incidence rate for the non-exposed group. "0.0" and "-0.0" represent rounding for values that are slightly greater and slightly less than zero, respectively.

^‡^As determined by the investigator.

**Table 4:** Summary of adverse events by system organ class

	Incidence Rate per 100 Patient-years^†^		
System Organ Class	Sitagliptin 100 mg	Non-exposed	Difference between Sitagliptin and Non-exposed (95% CI)*
Blood and Lymphatic System Disorders	1.1	0.6	0.4 (0.0, 0.8)
Cardiac Disorders	4.2	4.3	-0.2 (-1.1, 0.7)
Congenital, Familial, and Genetic Disorders	0.2	0.3	-0.0 (-0.3, 0.2)
Ear And Labyrinth Disorders	1.7	2.0	-0.4 (-1.0, 0.2)
Endocrine Disorders	0.3	0.5	-0.2 (-0.5, 0.1)
Eye Disorders	4.0	4.3	-0.2 (-1.1, 0.6)
Gastrointestinal Disorders	26.0	27.7	-1.2 (-3.7, 1.2)
General Disorders And Administration Site Conditions	8.8	9.3	-0.6 (-1.9, 0.8)
Hepatobiliary Disorders	1.3	1.1	0.2 (-0.3, 0.7)
Immune System Disorders	1.0	1.1	-0.1 (-0.6, 0.3)
Infections And Infestations	49.2	48.1	1.8 (-1.7, 5.3)
Injury, Poisoning And Procedural Complications	9.7	9.4	0.7 (-0.7, 2.1)
Investigations	15.1	15.7	-1.2 (-3.0, 0.6)
Metabolism And Nutrition Disorders	9.3	16.3	-6.8 (-8.5, -5.2)
Musculoskeletal And Connective Tissue Disorders	20.3	19.4	1.0 (-1.1, 3.0)
Neoplasms Benign, Malignant And Unspecified	2.2	1.7	0.6 (-0.0, 1.2)
Nervous System Disorders	15.4	15.5	-0.2 (-2.0, 1.6)
Pregnancy, Puerperium, and Perinatal Conditions	0.0	0.1	-0.0**
Psychiatric Disorders	4.6	4.7	0.0 (-0.9, 1.0)
Renal And Urinary Disorders	3.0	3.0	-0.1 (-0.8, 0.7)
Reproductive System And Breast Disorders	2.8	3.2	-0.3 (-1.1, 0.5)
Respiratory, Thoracic And Mediastinal Disorders	8.8	8.6	0.2 (-1.2, 1.4)
Skin And Subcutaneous Tissue Disorders	8.6	7.3	1.3 (0.1, 2.5)
Social Circumstances	0.0	0.1	-0.0**
Surgical and Medical Procedures	0.1	0.1	0.0**
Vascular Disorders	5.8	5.8	-0.2 (-1.2, 0.9)

CI = confidence interval

^† ^100 * (number of patients with ≥ 1 event/person years of follow-up time).

* Between-group difference and 95% CI based on stratified analysis. Positive differences indicate that the incidence rate for the sitagliptin group is higher than the incidence rate for the non-exposed group. "0.0" and "-0.0" represent rounding for values that are slightly greater and slightly less than zero, respectively.

** 95% CIs were not computed for events that occurred in fewer than 4 patients in both groups, because the CIs would necessarily have included 0.

The incidence rates of specific adverse events with at least 1 incident event per 100 patient-years in either group are listed in Table 5. The most commonly reported adverse events (i.e., ≥ 5 incident events per 100 patient-years in either group) were hypoglycemia, upper respiratory tract infection, diarrhea, nasopharyngitis, influenza, and headache. Of these events, nasopharyngitis and headache occurred more frequently in the sitagliptin group, although the 95% CI around the between-group differences included 0. Hypoglycemia, diarrhea, upper respiratory infection, and influenza occurred more frequently in the non-exposed group, with the 95% CIs around the between-group differences excluding 0 for hypoglycemia and diarrhea.

**Table 5:** Adverse events with at least 1 incident event per 100 patient-years in one or both groups

Adverse Event	Incidence Rate per 100 Patient-years^†^		
	Sitagliptin 100 mg	Non-exposed	Difference between Sitagliptin and Non-exposed (95% CI)*
**Gastrointestinal disorders SOC**			
Abdominal pain^‡^^	1.3	1.7	-0.5 (-1.0, 0.0)
Constipation	2.6	1.9	0.8 (0.1, 1.4)
Diarrhea^‡^	6.9	9.6	-2.3 (-3.6, -1.0)
Dyspepsia	2.0	1.6	0.4 (-0.1, 1.0)
Gastritis	1.2	1.5	-0.3 (-0.8, 0.2)
Gastroesophageal reflux disease	1.1	0.8	0.3 (-0.1, 0.8)
Nausea^‡^	3.0	3.8	-0.5 (-1.3, 0.3)
Toothache	1.2	1.3	-0.2 (-0.7, 0.3)
Vomiting^‡^	1.8	1.9	0.0 (-0.6, 0.6)
**General disorders and administration site conditions SOC**			
Fatigue	1.8	2.5	-0.6 (-1.3, -0.0)
Peripheral Edema	2.4	2.4	-0.0 (-0.7, 0.6)
**Infections and infestations SOC**			
Bronchitis	4.2	3.8	0.4 (-0.4, 1.3)
Cellulitis	0.8	1.0	-0.2 (-0.6, 0.2)
Gastroenteritis	2.0	1.9	0.1 (-0.5, 0.7)
Gastroenteritis Viral	1.0	1.0	0.0 (-0.4, 0.5)
Influenza	4.5	5.2	-0.7 (-1.7, 0.2)
Nasopharyngitis	7.7	7.0	0.9 (-0.3, 2.1)
Pharyngitis	1.5	1.4	0.1 (-0.4, 0.6)
Sinusitis	2.7	2.7	0.1 (-0.6, 0.8)
Upper respiratory tract infection	8.6	9.0	-0.3 (-1.6, 1.0)
Urinary tract infection	4.1	4.2	-0.2 (-1.1, 0.6)
Viral infection	1.1	0.9	0.2 (-0.2, 0.7)
**Injury, poisoning, and procedural complications SOC**			
Contusion	1.0	0.8	0.1 (-0.3, 0.5)
Muscle strain	0.9	1.0	-0.1 (-0.6, 0.3)
**Investigations SOC**			
ALT increased	1.5	1.4	0.1 (-0.4, 0.6)
AST increased	1.0	1.0	0.0 (-0.4, 0.4)
Blood glucose decreased	0.5	1.0	-0.5 (-0.9, -0.1)
Blood glucose increased	2.3	3.6	-1.3 (-2.1, -0.6)
Blood uric acid increased	1.0	0.8	0.2 (-0.2, 0.6)
Weight increased	0.8	1.0	-0.2 (-0.7, 0.2)
**Metabolism and nutrition disorders SOC**			
Hyperglycemia	1.2	1.4	-0.2 (-0.7, 0.3)
Hypoglycemia^‡^	5.2	12.1	-6.8 (-8.3, -5.5)
**Musculoskeletal and connective tissue disorders SOC**			
Arthralgia	3.4	3.7	-0.3 (-1.2, 0.5)
Back pain	4.3	4.1	0.1 (-0.8, 1.0)
Muscle spasms	1.2	1.5	-0.3 (-0.8, 0.2)
Musculoskeletal pain	1.6	1.6	-0.1 (-0.6, 0.5)
Myalgia	1.2	1.2	-0.0 (-0.5, 0.4)
Neck pain	0.7	1.0	-0.3 (-0.7, 0.1)
Osteoarthritis	1.6	1.1	0.5 (-0.0, 1.0)
Pain in extremity	2.8	2.1	0.7 (0.1, 1.4)
**Nervous system disorders SOC**			
Dizziness	2.8	2.7	0.1 (-0.6, 0.9)
Headache	5.8	5.6	0.4 (-0.7, 1.4)
Hypoesthesia	0.7	1.1	-0.4 (-0.8, 0.0)
Paraesthesia	1.0	1.2	-0.2 (-0.7, 0.3)
**Psychiatric disorders SOC**			
Anxiety	0.9	1.0	-0.1 (-0.5, 0.3)
Depression	1.4	1.2	0.3 (-0.2, 0.8)
Insomnia	1.5	1.4	0.0 (-0.5, 0.6)
**Respiratory, thoracic, and mediastinal disorders SOC**			
Cough	2.7	2.6	0.0 (-0.7, 0.7)
Oropharyngeal pain	1.3	1.2	0.1 (-0.4, 0.6)
**Skin and subcutaneous tissue disorders SOC**			
Rash	1.3	0.9	0.4 (-0.1, 0.8)
**Vascular disorders SOC**			
Hypertension	3.6	3.6	-0.1 (-1.0, 0.7)

CI = confidence interval;

^†^100 * (number of patients with ≥ 1 event/person years of follow-up time).

* Between-group difference and 95% CI based on stratified analysis. Positive differences indicate that the incidence rate for the sitagliptin group is higher than the incidence rate for the non-exposed group. "0.0" and "-0.0" represent rounding for values that are slightly greater and slightly less than zero, respectively.

^‡^For these events, see also Table 9 for the results of the predefined primary analysis which excludes data after initiation of glycemic rescue therapy.

^^^Abdominal pain includes abdominal pain, upper and lower abdominal pain, and abdominal and epigastric discomfort.

For specific adverse events reported in which the between-group difference in incidence rates was >0.2 incident events per 100 patient-years and for which the 95% CI around the between-group difference excluded 0, there were 5 specific adverse events reported at a higher incidence rate in the sitagliptin group and 7 reported at a higher incidence rate in the non-exposed group (Table 6). The largest between-group differences in incidence rates were observed for the adverse events of hypoglycemia (6.8) and diarrhea (2.3), which occurred with higher incidence rates in the non-exposed group, and for constipation (0.8), which occurred with a higher incidence rate in the sitagliptin group (discussed below). While the incidence rate of "protein urine present" was also higher in the sitagliptin group, when the adverse events of albuminuria, microalbuminuria, albumin urine present, protein urine present, and proteinuria were combined and assessed, the between-group difference (95% CI) was 0.1 per 100 patient-years (-0.2, 0.5). Additional analyses of renal safety included assessment of mean changes in serum creatinine over time as well as the proportions of patients whose serum creatinine met the PDLC criterion of 2 or more consecutive measurements with an increase from baseline of ≥ 0.3 mg/dL or of ≥ 50% (modified from Mehta et al [22]); results of these analyses showed no significant between-group differences.

**Table 6:** Adverse events for which the 95% CI around the difference in incidence rates excludes 0 and the between-group difference is >0.2 incident events per 100 patient-years

Adverse Event	Incidence Rate per 100 Patient-years^†^		
	Sitagliptin 100 mg	Non-exposed	Difference between Sitagliptin and Non-exposed (95% CI)*
**Sitagliptin > Non-exposed**			
Atrial fibrillation^‡^	0.4	0.2	0.3 (0.0, 0.6)
Constipation	2.6	1.9	0.8 (0.1, 1.4)
Protein urine present^^^	0.5	0.2	0.3 (0.0, 0.5)
Pain in extremity	2.8	2.1	0.7 (0.1, 1.4)
Dermatitis Contact	0.7	0.3	0.5 (0.1, 0.8)
**Non-exposed > Sitagliptin**			
Diarrhea^#^	6.9	9.6	-2.3 (-3.6, -1.0)
Fatigue	1.8	2.5	-0.6 (-1.3, -0.0)
Blood glucose decreased	0.5	1.0	-0.5 (-0.9, -0.1)
Blood glucose increased	2.3	3.6	-1.3 (-2.1, -0.6)
Blood triglycerides increased	0.5	0.8	-0.4 (-0.7, -0.0)
Hypoglycemia^#^	5.2	12.1	-6.8 (-8.3, -5.5)
Sinus headache	0.1	0.3	-0.3 (-0.5, -0.1)

CI = confidence interval

^†^100 * (number of patients with ≥ 1 event/person years of follow-up time).

* Between-group difference and 95% CI based on stratified analysis. Positive differences indicate that the incidence rate for the sitagliptin group is higher than the incidence rate for the non-exposed group. "0.0" and "-0.0" represent rounding for values that are slightly greater and slightly less than zero, respectively.

^‡^When atrial fibrillation and atrial flutter were combined, the between-group difference (95% CI) was 0.2 (-0.1, 0.5). Incidence rates for atrial flutter were 0.0 and 0.1 for the sitagliptin and the non-exposed groups, respectively, with a between-group difference [95% CI] of -0.1 (-0.3, -0.0).

^When albuminuria, microalbuminuria, albumin in urine present, protein urine present, and proteinuria were combined, the between-group difference (95% CI) was 0.1 (-0.2, 0.5).

^# ^For these events, see also Table 9 for the results of the predefined primary analysis which excludes data after initiation of glycemic rescue therapy.

The incidence rate of drug-related adverse events overall was higher in the non-exposed group compared with the sitagliptin group (Table 3), primarily due to a greater proportion of patients reported with hypoglycemia in the non-exposed group. Analyses of drug-related adverse events that occurred at an incidence rate of at least 0.2 incident events per 100 patient-years in either treatment group are in Table 7. In addition to hypoglycemia, the incidence rates of drug-related adverse events of abdominal pain, diarrhea, gastritis, weight increased, and paraesthesia were higher in the non-exposed group compared to the sitagliptin group, with the 95% CI around the between-group differences excluding 0 (Table 7). Constipation (discussed below) was the only drug-related adverse event with a higher incidence rate in the sitagliptin group (0.8) than in the non-exposed group (0.5), with the 95% CI around the between-group differences excluding 0.

**Table 7:** Adverse events considered to be related to study drug^‡ ^that occurred at an incidence rate of ≥ 0.2 incident events per 100 patient-years in one or both groups

Adverse Event	Incidence Rate per 100 Patient-years^†^		
	Sitagliptin 100 mg	Non-exposed	Difference between Sitagliptin and Non-exposed (95% CI)*
**Gastrointestinal disorders SOC**			
Abdominal discomfort	0.3	0.3	0.0 (-0.3, 0.3)
Abdominal distension	0.3	0.2	0.1 (-0.1, 0.3)
Abdominal pain	0.1	0.5	-0.5 (-0.8, -0.2)
Abdominal pain upper	0.5	0.8	-0.2 (-0.6, 0.1)
Constipation	0.8	0.5	0.4 (0.1, 0.8)
Diarrhea	2.4	4.5	-1.8 (-2.7, -1.0)
Dry mouth	0.1	0.3	-0.1 (-0.4, 0.1)
Dyspepsia	0.5	0.5	0.0 (-0.3, 0.3)
Flatulence	0.3	0.5	-0.1 (-0.4, 0.2)
Gastritis	0.1	0.5	-0.3 (-0.6, -0.1)
Gastroesophageal reflux disease	0.2	0.1	0.1 (-0.1, 0.3)
Nausea	1.3	1.8	-0.4 (-1.0, 0.1)
Vomiting	0.4	0.4	-0.0 (-0.3, 0.3)
**General disorders and administration site conditions SOC**			
Fatigue	0.6	0.7	-0.2 (-0.6, 0.2)
Peripheral Edema	0.4	0.6	-0.2 (-0.5, 0.1)
**Infections and infestations SOC**			
Upper respiratory tract infection	0.4	0.3	0.2 (-0.1, 0.5)
Urinary tract infection	0.3	0.1	0.1 (-0.1, 0.3)
**Investigations SOC**			
ALT increased	0.5	0.4	0.0 (-0.3, 0.3)
AST increased	0.3	0.4	-0.2 (-0.5, 0.1)
Blood creatine phosphokinase increased	0.2	0.1	0.1 (-0.0, 0.3)
Blood glucose decreased	0.3	0.5	-0.2 (-0.5, 0.0)
Blood glucose increased	0.3	0.5	-0.2 (-0.5, 0.1)
Blood uric acid increased	0.3	0.2	0.1 (-0.2, 0.3)
Creatinine renal clearance decreased	0.3	0.3	0.0 (-0.2, 0.2)
Glycosylated hemoglobin increased	0.1	0.2	-0.1 (-0.3, 0.1)
Weight decreased	0.2	0.1	0.1 (-0.1, 0.3)
Weight increased	0.2	0.5	-0.3 (-0.6, -0.0)
**Metabolism and nutrition disorders SOC**			
Decreased appetite	0.2	0.2	0.1 (-0.1, 0.3)
Hyperglycemia	0.1	0.2	-0.1 (-0.4, 0.0)
Hypoglycemia	3.5	7.5	-3.8 (-5.0, -2.8)
**Musculoskeletal and connective tissue disorders SOC**			
Arthralgia	0.2	0.2	-0.0 (-0.3, 0.2)
Muscle spasms	0.1	0.2	-0.1 (-0.3, 0.1)
Myalgia	0.1	0.2	-0.1 (-0.3, 0.1)
**Nervous system disorders SOC**			
Dizziness	0.6	0.5	0.1 (-0.2, 0.4)
Headache	1.2	1.1	0.1 (-0.4, 0.6)
Paraesthesia	0.1	0.3	-0.2 (-0.4, -0.0)
**Psychiatric disorders SOC**			
Insomnia	0.2	0.1	0.1 (-0.1, 0.3)
**Respiratory, thoracic, and mediastinal disorders SOC**			
Cough	0.2	0.1	0.0 (-0.2, 0.2)
**Skin and subcutaneous tissue disorders SOC**			
Pruritus	0.2	0.2	-0.0 (-0.3, 0.2)
Rash	0.4	0.2	0.2 (-0.0, 0.5)
Urticaria	0.1	0.2	-0.1 (-0.3, 0.1)
**Vascular disorders SOC**			
Hypertension	0.3	0.2	0.1 (-0.1, 0.3)

CI = confidence interval;

^†^100 * (number of patients with ≥ 1 event/person years of follow-up time).

* Between-group difference and 95% CI based on stratified analysis. Positive differences indicate that the incidence rate for the sitagliptin group is higher than the incidence rate for the non-exposed group. "0.0" and "-0.0" represent rounding for values that are slightly greater and slightly less than zero, respectively.

^‡^As determined by the investigator

The incidence rates of serious adverse events overall were similar in each treatment group (Table 3). Specific serious adverse events, irrespective of the relationship to study drug, that were reported with an incidence rate of at least 0.2 incident events per 100 patient-years in either treatment group are in Table 8. For myocardial ischemia, the 95% CI for the between-group difference in incidence rates excluded 0, favoring the sitagliptin group. There were no other notable between-group differences.

**Table 8:** Serious adverse events irrespective of relationship to study drug that occurred at an incidence rate of ≥ 0.2 incident events per 100 patient-years in one or both groups

CI = confidence interval Adverse Event	Incidence Rate per 100 Patient-years^†^		
	Sitagliptin 100 mg	Non-exposed	Difference between Sitagliptin and Non-exposed (95% CI)*
**Cardiac disorders SOC**			
Acute myocardial infarction	0.1	0.2	-0.1 (-0.3, 0.0)
Angina pectoris	0.2	0.1	0.1 (-0.1, 0.3)
Coronary artery disease	0.2	0.4	-0.2 (-0.5, 0.0)
Myocardial infarction	0.2	0.2	0.0 (-0.2, 0.2)
Myocardial ischemia	0.0	0.2	-0.2 (-0.4, -0.1)
**General disorders and administration site conditions SOC**			
Non-cardiac chest pain	0.1	0.3	-0.1 (-0.4, 0.1)
**Infections and Infestations SOC**			
Pneumonia	0.2	0.2	0.1 (-0.2, 0.3)
**Neoplasms benign, malignant and unspecified SOC**			
Basal cell carcinoma	0.2	0.2	0.0 (-0.2, 0.2)
Breast cancer^‡^	0.3	0.2	0.1 (-0.2, 0.5)
Prostate cancer^‡^	0.2	0.2	-0.0 (-0.3, 0.3)
**Nervous system disorders SOC**			
Cerebrovascular accident	0.1	0.2	-0.1 (-0.3, 0.1)
Transient ischemia attack	0.0	0.2	-0.1 (-0.3, 0.0)

CI = confidence interval

^†^100 * (number of patients with ≥ 1 event/person years of follow-up time).

* Between-group difference and 95% CI based on stratified analysis. Positive differences indicate that the incidence rate for the sitagliptin group is higher than the incidence rate for the non-exposed group. "0.0" and "-0.0" represent rounding for values that are slightly greater and slightly less than zero, respectively.

^‡ ^Gender-specific analyses for these adverse events. There were no reports of breast cancer in males.

### Adverse Events of Interest

#### Hypoglycemia

The incidence rates of hypoglycemia were based upon symptomatic reports of hypoglycemia, regardless of a concurrent glucose measurement, although a majority (73.9%) of events (including incident events and subsequent events) was confirmed by a (fingerstick) glucose of ≤ 70 mg/dL. The predefined primary analysis for hypoglycemia (i.e., excluding data after initiation of glycemic rescue therapy) showed a between-group difference (95% CI) of -6.7 incident events per 100 patient-years (-8.2, -5.3), favoring the sitagliptin group (Table 9). The difference observed for hypoglycemia was mainly due to the use of a sulfonylurea as a comparator agent in 2 studies of up to 2 years in duration [11,23,24], as well as a study in which patients were switched from placebo to a sulfonylurea during a double-blind continuation period (P020 in Table 1). However, results from some individual studies included in this pooled analysis (in which sitagliptin was added to either a sulfonylurea with or without metformin [25] or to insulin with or without metformin [26]) demonstrated an increased risk for hypoglycemia with sitagliptin relative to placebo. Therefore, a separate pooled analysis of hypoglycemia was conducted in which confounding effects of a sulfonylurea or insulin as either background or comparator therapy were removed. In this analysis, which compares sitagliptin to either placebo or AHAs not known to increase rates of hypoglycemia (i.e., metformin or a thiazolidinedione), the incident rates of hypoglycemia were 3.1 and 3.3 per 100 patient-years in the sitagliptin (n = 4175) and non-exposed (n = 3572) groups, respectively.

**Table 9:** Select gastrointestinal and hypoglycemia adverse events: Predefined primary analysis, which excluded data after initiation of glycemic rescue therapy

Adverse Event	Incidence Rate per 100 Patient-years^†^		
	Sitagliptin 100 mg	Non-exposed	Difference between Sitagliptin and Non-exposed (95% CI)*
**Gastrointestinal disorders SOC**			
One or more select event (abdominal pain^‡^, diarrhea, nausea, vomiting)	14.0	17.2	-2.9 (-4.8, -1.1)
Abdominal pain^‡^	4.1	4.7	-0.7 (-1.7, 0.3)
Diarrhea	7.1	10.0	-2.5 (-3.9, -1.1)
Nausea	3.1	4.0	-0.7 (-1.6, 0.2)
Vomiting	1.9	1.9	0.0 (-0.6, 0.6)
**Metabolism and nutrition disorders SOC**			
Hypoglycemia	4.9	11.7	-6.7 (-8.2, -5.3)

CI = confidence interval;

^†^100 * (number of patients with ≥ 1 event/person years of follow-up time).

* Between-group difference and 95% CI based on stratified analysis. Positive differences indicate that the incidence rate for the sitagliptin group is higher than the incidence rate for the non-exposed group. "0.0" and "-0.0" represent rounding for values that are slightly greater and slightly less than zero, respectively.

^‡^Abdominal pain includes abdominal pain, upper and lower abdominal pain, and abdominal and epigastric discomfort.

#### Gastrointestinal Symptoms

The predefined primary analysis (i.e., excluding data after initiation of glycemic rescue therapy) of select GI adverse events demonstrated lower incidence rates for the pooled select terms and for the specific adverse event of diarrhea in the sitagliptin group (Table 9). The differences observed for diarrhea mainly reflected the use of metformin as a comparator; when the confounding effects of initiation of metformin were removed, the incidence rates were 4.9 and 5.0 per 100 patient-years in the sitagliptin (n = 3904) and non-exposed (n = 3310) groups, respectively. There were no meaningful differences between treatment groups in the incidence rates for the specific events of nausea or vomiting, or the composite abdominal pain events (Table 9). The incidence rate of the adverse event of constipation was higher in the sitagliptin group (2.6) than in the non-exposed group (1.9) (Table 6); the majority of events were mild in intensity, with 1 patient in each treatment group discontinuing treatment due to the adverse event.

#### Cardiovascular

The incidence rates of adverse events in the Cardiac Disorders SOC were similar between groups (Table 4). In a prespecified MACE analysis (see Appendix I for listing of MACE terms included in the analysis), there was a total of 64 patients for whom at least 1 MACE-related adverse event was reported. The incidence rates were 0.6 per 100 patient-years in the sitagliptin group and 0.9 in the non-exposed group, for a between-group difference (95% CI) of -0.3 (-0.7, 0.1). In the prespecified analysis, the risk ratio for sitagliptin-exposed relative to non-exposed patients was 0.68 (95% CI: 0.41, 1.12). In a sensitivity analysis using the Mantel-Haenszel approach, the results were consistent (risk ratio [95% CI] = 0.67 [0.39, 1.13]).

#### Malignancy

The incidence rates of adverse events in the Neoplasms SOC overall were 2.2 per 100 patient-years in the sitagliptin-exposed group and 1.7 in the non-exposed group (between-group difference [95% CI] of 0.6 [-0.0, 1.2]). The analysis of the combined events of malignancies within the Neoplasms SOC showed an incidence rate of 1.0 for both the sitagliptin and non-exposed groups (Table 10). Low incidence rates of a wide range of specific malignancies were reported with similar frequencies in both treatment groups. The most frequently reported events were basal cell carcinoma (0.2 per 100 patient-years in each group, with a between-group difference [95% CI] of 0.0 [-0.2, 0.2]), breast cancer (in women, 0.3 and 0.2 in the sitagliptin and non-exposed groups, respectively, with a between-group difference [95% CI] of 0.1 [-0.2, 0.5]) and prostate cancer (in men, 0.2 in each group, with a between-group difference [95% CI] of -0.0 [-0.3, 0.3]) (Table 8). Results from a sensitivity analysis excluding events of non-melanomatous skin cancers showed similar between-group incidence rates: 0.8 in each group, with a between-group difference (95% CI) of -0.1 (-0.5, 0.3).

**Table 10:** Any malignancy adverse events

Adverse Event	n/patient-years of exposure (Incidence Rate per 100 Patient-years^†^)		
	Sitagliptin 100 mg	Non-exposed	Difference between Sitagliptin and Non-exposed (95% CI)*
Any malignancy	46/4690 (1.0)	40/3930 (1.0)	-0.0 (-0.5, 0.4)

n = number of patients with ≥ 1 occurrence of the endpoint; CI = confidence interval;

^†^100 * (number of patients with ≥ 1 event/person years of follow-up time).

* Between-group difference and 95% CI based on stratified analysis. Positive differences indicate that the incidence rate for the sitagliptin group is higher than the incidence rate for the non-exposed group. "0.0" and "-0.0" represent rounding for values that are slightly greater and slightly less than zero, respectively.

After excluding malignancies, a higher incidence rate for the combined residual terms of non-malignant adverse events within the Neoplasms SOC was observed in the sitagliptin group compared with the non-exposed group (between-group difference [95% CI] of 0.6 [0.2, 1.1]; Additional file 1: Supplemental Table S1). This difference was not the result of an imbalance in any single adverse event or any group of biologically-related adverse events. Among these residual adverse events, the most frequently reported were uterine leiomyoma/leiomyoma (in women, 0.5 and 0.2 in the sitagliptin and the non-exposed groups, respectively; between group difference [95% CI] of 0.4 [-0.0, 0.9]) and lipoma (0.2 and 0.1 in the sitagliptin and the non-exposed groups, respectively; between-group difference [95% CI] of 0.1 [-0.0, 0.3]). Among adverse event terms within the Neoplasms SOC, uterine leiomyoma was the most common condition reported as part of patient medical history (i.e., present prior to randomization) in 3.0% and 2.8% of randomized women in the sitagliptin and non-exposed groups, respectively, while lipoma was reported as part of medical history in 0.3% and 0.4% of randomized patients in the sitagliptin and non-exposed groups, respectively.

To further explore the small between-group difference in the incidence rate for the residual, non-malignant adverse events overall within the Neoplasms SOC, analyses were conducted in the following additional populations: (1) a broader patient population (N = 6748 and 4855 patients in the sitagliptin and non-exposed groups, respectively) consisting of the primary population plus patients dosed with sitagliptin other than 100 mg daily (i.e., 12.5, 50, and 200 mg daily) or comparator, including patients with renal insufficiency who were randomized to receive dose-adjusted sitagliptin or comparator, and (2) the subset of this broader patient population randomized to receive the higher dose of sitagliptin (200 mg once daily; N = 456) or comparator (N = 363) for up to 1 year. These analyses showed results similar to those observed in the primary analysis, with between-group differences (95% CI) in the overall incidence rate for the residual non-malignant adverse events within the Neoplasms SOC of 0.7 (0.3, 1.2) and 0.6 (-2.6, 3.4) for the broader population and the sitagliptin 200-mg population, respectively. Additional analyses of residual non-malignant adverse events within the Neoplasms SOC combined with neoplasms that MedDRA assigns to other SOCs (e.g., polyps) were consistent with findings limited to analysis of the residual non-malignant adverse events within the Neoplasms SOC.

#### Bone Fracture

In the primary analysis of bone fracture, which excluded fractures of the face and skull and those related to high-impact trauma, the incidence rates of bone fractures were 0.8 per 100 patient-years in the sitagliptin group and 1.0 in the non-exposed group (between-group difference [95% CI] = -0.1 [-0.6, 0.3]). Results were similar to the above when the potentially confounding influence of initiation of a thiazolidinedione was eliminated. The incidence rate of overall bone fractures, including fractures of the face and skull and those related to high-impact trauma, was 1.1 in each treatment group (between-group difference [95% CI] = 0.1 [-0.4, 0.5]).

#### Infection

The incidence rates for infections overall and for specific adverse events of infection were generally similar between groups (Tables 4 and 5). A sensitivity analysis that included all patients with events of upper respiratory tract infection or related terms (including viral upper respiratory tract infection, upper respiratory tract infection bacterial, and upper respiratory fungal infection) demonstrated no meaningful between-group difference (-0.5 per 100 patient-years [95% CI: -1.9, 0.8]). A similar sensitivity analysis conducted for the adverse event of bronchitis (including all patients with events in the Infections and Infestations SOC that contained the term "bronchitis") also showed no meaningful between-group difference (0.5 [95% CI: -0.4, 1.4]). The incidence rate of the generally more severe, respiratory-specific adverse event of pneumonia was similar in both treatment groups (0.8 in each treatment group; between-group difference [95% CI] = 0.1 [-0.3, 0.5]).

For the adverse event of urinary tract infection, the incidence rates were balanced between the sitagliptin and the non-exposed groups (Table 5). Similar incidence rates for both groups were also observed when infection events of cystitis were pooled with combined events of urinary tract infection (including urinary tract infection, Escherichia urinary tract infection, and urinary tract infection, bacterial): 4.5 and 4.8 per 100 patient-years in the sitagliptin and the non-exposed groups, respectively, with a between-group difference of -0.3 (95% CI: -1.3, 0.6). Incidence rates of the generally more severe infection of pyelonephritis (including pyelonephritis, pyelonephritis acute, and pyelonephritis chronic) were generally similar between the two treatment groups: 0.1 and 0.2 in the sitagliptin and non-exposed groups, respectively, with a between-group difference of -0.1 (95% CI: -0.3, 0.1).

The incidences of other events potentially suggestive of an immunodeficiency, due to either severity or due to the specific nature of the infection, were also examined. There was a lower incidence rate of the adverse event of sepsis/Staphylococcal sepsis (all reported as serious) in the sitagliptin group (1 patient, 0.0 per 100 patient-years) relative to the non-exposed group (4 patients, 0.1). The overall incidence rates of adverse experiences of abscesses (including any term containing abscess) were similar for the sitagliptin group (1.2) relative to the non-exposed group (1.3), with a between-group difference of -0.1 (95% CI: -0.6, 0.4). The incidence rates for combined events of herpes infections (including genital herpes, herpes ophthalmic, herpes simplex, herpes viral infection, herpes zoster, and oral herpes) were similar in the sitagliptin group (0.8) and the non-exposed group (0.9), with a between-group difference of -0.0 (95% CI: -0.4, 0.4). There was no increase in recrudescence of infection (i.e., herpes zoster), with incidence rates of 0.5 and 0.6 in the sitagliptin and non-exposed groups, respectively (between-group difference of -0.1 [95% CI: -0.4, 0.2]). For all these events, patients treated with sitagliptin generally had no greater frequency, severity, or duration of events relative to patients not treated with sitagliptin. There was no signal for an increased risk of opportunistic infections in patients treated with sitagliptin.

#### Angioedema

At baseline, 31% of patients reported taking an ACE inhibitor, and approximately 34% were on an ACE inhibitor at any time during the clinical studies (for a total exposure of 1,532 and 1,251 patient-years on an ACE inhibitor for the sitagliptin and non-exposed groups, respectively). In this analysis, 90 incident angioedema-related events were reported, of which 31 occurred in patients while on an ACE inhibitor. The incidence rates of angioedema-related events while on an ACE inhibitor were similar in the sitagliptin and non-exposed groups (1.0 and 1.3 per 100 patient-years, respectively). The incidence rate while not on an ACE inhibitor was 1.1 in each group. For the specific adverse event of angioedema, the incidence rates were 0.06 and 0.08 while on an ACE inhibitor and 0.03 and 0.04 while not on an ACE inhibitor in the sitagliptin and non-exposed groups, respectively.

#### Skin

As described earlier, a few adverse events accounted for the modest difference in incidence rates between groups within the Skin and Subcutaneous Disorders SOC. Of the adverse events within this SOC, the MedDRA preferred term of rash was the only skin-related adverse event with at least 1 incident event per 100 patient-years in either group (Table 5). No adverse events of Stevens-Johnson syndrome, 1 event of erythema multiforme (for a patient in the non-exposed group), and 1 event of leukocytoclastic vasculitis (for a patient in the sitagliptin group) were reported.

#### Liver Function Tests

The incidence rates of adverse events of increased ALT or AST were similar between groups (Table 5). Further, the proportions of patients in the sitagliptin and non-exposed groups with their last measurement (obtained either at time of discontinuation or at the final scheduled study visit) of ALT ≥ 3 times the upper limit of normal were 0.8% and 0.7%, respectively, with a between-group difference (95% CI) of 0.2% (-0.2, 0.5); the proportions with last AST measurement ≥ 3 times the upper limit of normal were 0.3% and 0.3%, respectively, with a between-group difference (95% CI) of -0.0% (-0.3, 0.2).

## Discussion

In this updated pooled analysis of 19 double-blind, randomized clinical studies up to 2 years in duration, treatment with the DPP-4 inhibitor, sitagliptin, was generally well tolerated, with exposure-adjusted incidence rates of adverse events generally similar to those observed with other treatments. This updated analysis includes data from additional treatment regimens and extends the findings of a previously published analysis [1] by including results from an additional 4,100 patients, thereby increasing the pooled population from 6,139 to 10,246 patients and the total exposure from ~5,300 to ~8,700 patient-years. Overall, the present findings based on the substantially larger dataset were generally consistent with those from the previously published pooled analysis of 12 clinical studies.

In the present pooled analysis, the incidence rate of hypoglycemia was lower in sitagliptin-treated patients compared to those not treated with sitagliptin. This difference was mainly due to use of a sulfonylurea as a comparator agent in some studies. In clinical trials, the incidence of hypoglycemia with sitagliptin has been similar to placebo when sitagliptin is used as monotherapy, as initial combination therapy with metformin or pioglitazone, or as add-on therapy to agents that are not by themselves associated with hypoglycemia (e.g., metformin or a thiazolidinedione) [27-29]. However, when sitagliptin is added to ongoing therapy with a sulfonylurea or insulin, there is an increase in the incidence of hypoglycemia compared with the addition of placebo as might be expected when an agent that provides additional glycemic efficacy is used in combination with these agents [25,26]. Thus, the reported incidence rate of hypoglycemia in the sitagliptin group reflects the combined rate across multiple studies with different treatment regimens. Clinicians should review the hypoglycemia rates from studies with specific sitagliptin-based regimens to understand the potential risks for their patients.

GLP-1 receptor agonists and certain oral AHAs are associated with an increased risk of specific GI side effects [4,30]. In the present pooled analysis, the incidences of GI adverse events overall were similar in the sitagliptin and the non-exposed groups. There was a small increase in incidence rate (an increase of 0.8 per 100 patient-years) for constipation in sitagliptin-treated patients and a higher incidence rate of diarrhea in non-exposed patients. The increase in diarrhea in the non-exposed group was due to the use of metformin as a comparator agent in some trials. The reason for the increased incidence of constipation in the sitagliptin group is not known. While the higher levels of endogenous GLP-1 achieved with DPP-4 inhibitors do not appear to slow gastric emptying [31,32], increased levels of endogenous GLP-1 may have some effect on gut motility [33].

Prior meta-analyses of published studies of DPP-4 inhibitors (including sitagliptin and vildagliptin) have reported an increased risk for infections overall and for specific infections (nasopharyngitis, upper respiratory tract infection, and urinary tract infection) [9,26,34]. In the present analysis, however, no notable between-group differences in incidence rates were observed for any infection-related adverse events, including nasopharyngitis, upper respiratory tract infection, and urinary tract infection. The larger patient population and access to patient-level data in the present analysis, allowing for a more-detailed assessment of these adverse events, likely account for the differences between the present and aforementioned findings.

An increased risk of bone loss and fracture has been reported in patients with type 2 diabetes treated with thiazolidinediones [6,7]. The present analysis found that patients treated with sitagliptin had a similar incidence rate of bone fracture relative to those not exposed to sitagliptin. The clinical results with sitagliptin are consistent with those from a study in an animal model of osteoporosis [35]. In ovariectomized rats, treatment with sitagliptin had no effect on bone mass relative to vehicle treatment, whereas treatment with thiazolidinediones exacerbated bone loss.

Patients with type 2 diabetes are at an increased risk for cardiovascular morbidity and mortality [36]. This increased risk is thought to be related to the high rate of co-morbidities, such as hypertension and dyslipidemia, as well as the potential effect of hyperglycemia. However, interest in the effect of specific antihyperglycemic therapies on cardiovascular outcomes, which was first raised as a result of the University Group Diabetes Program [37], has resurfaced subsequent to a meta-analysis that indicated an increased risk for cardiovascular events with rosiglitazone [38]. These and other studies have led to an increased focus on the cardiovascular risk associated with individual antihyperglycemic agents used to treat diabetes.

In the present analysis, in which 82% of patients had cardiovascular risk factors in addition to type 2 diabetes and cardiovascular disease, there was no difference between groups in the evaluation of the Cardiac Disorders SOC overall. Further, a MACE analysis focused on ischemic events revealed that there were 0.6 incident events per 100 patient-years in the sitagliptin group and 0.9 in the non-exposed group (incidence rate ratio [sitagliptin/non-exposed] = 0.68 [95% CI: 0.41, 1.12]). These results suggest that sitagliptin does not increase cardiovascular risk in patients with type 2 diabetes. The impact of sitagliptin on cardiovascular outcomes when used as part of usual care will be compared with the impact of usual care without sitagliptin in the ongoing, randomized, placebo-controlled Trial Evaluating Cardiovascular Outcomes with Sitagliptin (TECOS) [39], which is planned to enroll 14,000 patients age 50 years or older with type 2 diabetes and documented cardiovascular disease. The results from this study will provide a comprehensive cardiovascular assessment of patients treated with sitagliptin relative to those not treated with sitagliptin.

Both obesity and diabetes are reported to be associated with an increased risk of malignancy, potentially related to the growth-promoting effects of hyperinsulinemia associated with insulin resistance [40]. In this context, the impact of antihyperglycemic therapies on the risk of malignancy has recently been the focus of increasing attention [3]. In the current pooled dataset, the analysis of adverse events within the Neoplasms SOC overall, which includes all adverse event terms for malignant neoplasms as well as residual terms for a wide variety of non-malignant neoplasms, showed a low overall incidence rate of adverse events in each treatment group. No differences between groups were observed in the incidence rate of malignancies overall or in the incidence rates of any specific malignancy. A slightly higher incidence rate of adverse events within the Neoplasms SOC overall observed in the sitagliptin group was due to reports of different types of non-malignant neoplasms. Of these, the specific events that were most commonly reported were lipoma and uterine leiomyoma, consistent with their frequent occurrence in the general adult population [41,42]. Results of additional analyses, including analysis of a broader patient population and of a population of patients exposed to the higher 200-mg dose of sitagliptin, were not suggestive of a dose-dependent relationship to explain these findings.

Since the residual, non-malignant adverse events in the Neoplasms SOC included a collection of disparate and diverse types of lesions of varying histology and biology, the ability to interpret aggregate data from this type of categorization may be limited. Thus, while an occult bias leading to increased ascertainment of events in the sitagliptin group can not be ruled out, the large number of unrelated adverse event terms assessed in these pooled analyses and the varying and diverse histologies that underlie the reported neoplasms suggest that the small increase in the incidence rate of non-malignant neoplasms in the sitagliptin group relative to the non-exposed group may be a stochastic finding and not related to the use of sitagliptin.

Skin-related adverse events were of interest, since administration of some DPP-4 inhibitors [43,44], but not sitagliptin [45], was associated with dose-dependent necrotic skin lesions in preclinical studies in monkeys and dogs. However, skin findings consistent with these preclinical lesions have not been reported in patients in controlled clinical studies with DPP-4 inhibitors, including those contributing to the present pooled analysis for sitagliptin. In the present analysis, the increased incidence rate of skin-related adverse events overall observed in the sitagliptin group was due to small differences in a few adverse events, including contact dermatitis and rash.

Reduced DPP-4 enzyme activity and DPP-4 enzyme deficiency have been associated with an increase in ACE inhibitor-induced angioedema in rats and humans [46,47], and it was hypothesized that concomitant use of DPP-4 inhibitors and ACE inhibitors could increase the risk of angioedema-related adverse events. This hypothesis was supported by an analysis of another DPP-4 inhibitor [8]. However, a higher rate of angioedema or angioedema-related events was not observed in the present analysis in sitagliptin-treated patients compared to patients not exposed to sitagliptin, regardless of ACE inhibitor use.

The following are limitations of the present pooled analysis: the results are from patients included in randomized, controlled clinical studies of up to 2 years in duration and, thus, may not be fully reflective of use in the general population; the analysis focused on sitagliptin 100 mg/day, the usual clinical dose; and there were multiple comparisons made without an adjustment for multiplicity, which increased the chance for spurious findings. The strengths of these analyses include: the ability to account for all reported adverse events using patient-level data; the large number of clinical trials and patients analyzed; and the sensitivity analyses supporting the robustness of the findings.

## Conclusions

In this updated pooled safety analysis based upon data available as of July 2009 from over 10,000 patients with type 2 diabetes, treatment with sitagliptin 100 mg/day was generally well tolerated as monotherapy, as initial combination therapy, and as add-on therapy in double-blind, randomized clinical studies of up to 2 years in duration. Continued assessment of adverse events reported from clinical trials and from the post-marketing environment is ongoing.

## Competing interests

All authors are employed by Merck Sharp & Dohme, Corp., a subsidiary of Merck & Co., Inc., the manufacturer of sitagliptin and may have company stock or stock options.

## Authors' contributions

DWH, SSE, ER, GTG, HG, BJM, KDK, and BJG conceived the design for the analyses. GTG, HG, and BJM performed the statistical analyses. All authors were involved in the interpretation of the analyses. All authors were involved in drafting the manuscript or revising it critically for important intellectual content. All authors approved the final manuscript.

## Appendix I

The prespecified MedDRA terms for the MACE analysis

Acute myocardial infarction*

Basal ganglia infarction

Basilar artery thrombosis

Brain stem infarction

Brain stem stroke

Brain stem thrombosis

Carotid arterial embolus

Carotid artery thrombosis

Cerebellar artery thrombosis

Cerebellar embolism

Cerebellar infarction*

Cerebral artery embolism

Cerebral artery thrombosis

Cerebral infarction*

Cerebral thrombosis

Cerebrovascular accident*

Coronary artery thrombosis

Coronary bypass thrombosis

Embolic cerebral infarction

Embolic stroke

Hemorrhagic cerebral infarction

Hemorrhagic stroke*

Hemorrhagic transformation stroke

Ischemic cerebral infarction

Ischemic stroke*

Lacunar infarction*

Lateral medullary syndrome

Moyamoya disease

Myocardial infarction*

Papillary muscle infarction

Post procedural myocardial infarction

Post procedural stroke

Silent myocardial infarction*

Stroke in evolution

Thalamic infarction*

Thrombotic cerebral infarction

Thrombotic stroke

Wallenberg syndrome

MACE = major adverse cardiovascular events

Terms marked with an asterisk (*) were observed in the pooled data set.

Additionally, all deaths determined to be potentially cardiovascular-related (based on blinded clinical review) were included in the MACE analysis.

## Appendix II

Expanded Standard MedDRA Query (SMQ) terms for angioedema

Allergic edema*

Anaphylactic reaction*,**

Angioedema*

Circumoral edema

Conjunctival edema

Corneal edema

Drug hypersensitivity*,**

Edema mouth

Epiglottic edema

Eye edema

Eye swelling*

Eyelid edema*

Face edema*

Gingival edema

Gingival swelling*

Gleich's syndrome

Hereditary angioedema

Hypersensitivity*,**

Idiopathic urticaria

Laryngeal edema

Laryngotracheal edema

Lip edema

Lip swelling*

Oculorespiratory syndrome

Oropharyngeal swelling

Palatal edema*

Periorbital edema*

Pharyngeal edema

Scleral edema

Small bowel angioedema

Swelling face*

Swollen tongue*

Tongue edema

Tracheal edema

Urticaria*

Urticaria cholinergic

Urticaria chronic

Urticaria popular

*This adverse event was observed in the pooled data set.

**Additional term included with SMQ.

## Pre-publication history

The pre-publication history for this paper can be accessed here:

http://www.biomedcentral.com/1472-6823/10/7/prepub

## Supplementary Material

Additional file 1 **Supplemental Table S1. Non-malignant adverse events in the Neoplasm SOC**. The table lists the types and incidence rates of the non-malignant adverse events. Click here for file

## References

[B1] Williams-Herman D Round E Swern AS Musser B Davies MJ Stein PP Safety and tolerability of sitagliptin in patients with type 2 diabetes: a pooled analysis BMC Endocr Disord 2008 8 14 10.1186/1472-6823-8-14 18954434 PMC2605739

[B2] McGuire DK Inzucchi SE New drugs for the treatment of diabetes mellitus: part I: Thiazolidinediones and their evolving cardiovascular implications Circulation 2008 117 440 449 10.1161/CIRCULATIONAHA.107.704080 18212301

[B3] Smith U Gale EA Does diabetes therapy influence the risk of cancer? Diabetologia 2009 52 1699 1708 10.1007/s00125-009-1441-5 19597799

[B4] Inzucchi SE Oral antihyperglycemic therapy for type 2 diabetes: scientific review JAMA 2002 287 360 372 10.1001/jama.287.3.360 11790216

[B5] Inzucchi SE McGuire DK New drugs for the treatment of diabetes: part II: Incretin-based therapy and beyond Circulation 2008 117 574 584 10.1161/CIRCULATIONAHA.107.735795 18227398

[B6] Dormuth CR Carney G Carleton B Bassett K Wright JM Thiazolidinediones and fractures in men and women Arch Intern Med 2009 169 1395 1402 10.1001/archinternmed.2009.214 19667303

[B7] Meier C Kraenzlin ME Bodmer M Jick SS Jick H Meier CR Use of thiazolidinediones and fracture risk Arch Intern Med 2008 168 820 825 10.1001/archinte.168.8.820 18443256

[B8] Brown NJ Byiers S Carr D Maldonado M Warner BA Dipeptidyl peptidase-IV inhibitor use associated with increased risk of ACE inhibitor-associated angioedema Hypertension 2009 54 516 523 10.1161/HYPERTENSIONAHA.109.134197 19581505 PMC2758288

[B9] Amori RE Lau J Pittas AG Efficacy and safety of incretin therapy in type 2 diabetes: systematic review and meta-analysis JAMA 2007 298 194 206 10.1001/jama.298.2.194 17622601

[B10] Monami M Iacomelli I Marchionni N Mannucci E Dipeptydil peptidase-4 inhibitors in type 2 diabetes: A meta-analysis of randomized clinical trials Nutr Metab Cardiovasc Dis 2009 in press 10.1016/j.numecd.2009.03.015 19515542

[B11] Scott R Wu M Sanchez M Stein P Efficacy and tolerability of the dipeptidyl peptidase-4 inhibitor sitagliptin as monotherapy over 12 weeks in patients with type 2 diabetes Int J Clin Pract 2007 61 171 180 10.1111/j.1742-1241.2006.01246.x 17156104

[B12] Hanefeld M Herman GA Wu M Mickel C Sanchez M Stein PP Once-daily sitagliptin, a dipeptidyl peptidase-4 inhibitor, for the treatment of patients with type 2 diabetes Curr Med Res Opin 2007 23 1329 1339 10.1185/030079907X188152 17559733

[B13] Goldstein BJ Feinglos MN Lunceford JK Johnson J Williams-Herman DE Effect of initial combination therapy with sitagliptin, a dipeptidyl peptidase-4 inhibitor, and metformin on glycemic control in patients with type 2 diabetes Diabetes Care 2007 30 1979 1987 10.2337/dc07-0627 17485570

[B14] Reasner CA Olansky L Seck T Williams-Herman D Luo E Chen M Initial therapy with the fixed-dose combination (FDC) if sitagliptin and metformin (JANUMET) in patients with type 2 diabetes mellitus provides superior glycaemic control and HbA_1c _goal attainment with lower rates of abdominal pain and diarrhea vs metformin alone (abstract) Diabetologia 2009 52 Suppl 1 S295

[B15] Olansky L Reasner CA Seck T Williams-Herman D Luo E Chen M A strategy implementing initial therapy with a fixed-dose combination tablet of sitagliptin and metformin in patients with type 2 diabetes provides superior glycemic control compared with a strategy using initial metformin monotherapy over 44 weeks (abstract) IDF Abstract Book 2009 0-0535 178

[B16] Chan JC Scott R Arjona Ferreira JC Sheng D Gonzalez E Davies MJ Safety and efficacy of sitagliptin in patients with type 2 diabetes and chronic renal insufficiency Diabetes Obes Metab 2008 10 545 555 10.1111/j.1463-1326.2008.00914.x 18518892

[B17] Guidance for Industry: Collection of race and ethnicity data in clinical trials. 9/2/2005 http://www.fda.gov/downloads/Drugs/GuidanceComplianceRegulatoryInformation/Guidances/ucm071596.pdf (last accessed February 9, 2010).

[B18] Miettinen O Nurminen M Comparative analysis of two rates Stat Med 1985 4 213 226 10.1002/sim.4780040211 4023479

[B19] Engel SS Williams-Herman DE Golm GT Clay RJ Machotka SV Kaufman KD Sitagliptin: review of preclinical and clinical data regarding incidence of pancreatitis Int J Clin Pract in press 20412332 10.1111/j.1742-1241.2010.02382.x PMC2904489

[B20] Breslow NE Day NE Statistical methods in cancer research. Volume II--The design and analysis of cohort studies IARC Sci Publ 1987 1 406 3329634

[B21] Robins J Breslow N Greenland S Estimators of the Mantel-Haenszel variance consistent in both sparse data and large-strata limiting models Biometrics 1986 42 311 323 10.2307/2531052 3741973

[B22] Mehta RL Kellum JA Shah SV Molitoris BA Ronco C Warnock DG Acute Kidney Injury Network: report of an initiative to improve outcomes in acute kidney injury Crit Care 2007 11 R31 10.1186/cc5713 17331245 PMC2206446

[B23] Nauck MA Meininger G Sheng D Terranella L Stein PP Efficacy and safety of the dipeptidyl peptidase-4 inhibitor, sitagliptin, compared with the sulfonylurea, glipizide, in patients with type 2 diabetes inadequately controlled on metformin alone: a randomized, double-blind, non-inferiority trial Diabetes Obes Metab 2007 9 194 205 10.1111/j.1463-1326.2006.00704.x 17300595

[B24] Seck T Nauck MA Sheng D Sunga S Davies MJ Stein PP Safety and efficacy of treatment with sitagliptin or glipizide in patients with type 2 diabetes inadequately controlled on metformin: A 2-year study Int J Clin Pract 2010 64 562 576 10.1111/j.1742-1241.2010.02353.x 20456211

[B25] Hermansen K Kipnes M Luo E Fanurik D Khatami H Stein P Efficacy and safety of the dipeptidyl peptidase-4 inhibitor, sitagliptin, in patients with type 2 diabetes mellitus inadequately controlled on glimepiride alone or on glimepiride and metformin Diabetes Obes Metab 2007 9 733 745 10.1111/j.1463-1326.2007.00744.x 17593236

[B26] Vilsboll T Rosenstock J Yki-Jarvinen H Cefalu WT Chen Y Luo E Efficacy and safety of sitagliptin when added to insulin therapy in patients with type 2 diabetes Diabetes Obes Metab 2010 12 167 177 10.1111/j.1463-1326.2009.01173.x 20092585

[B27] Karasik A Aschner P Katzeff H Davies MJ Stein PP Sitagliptin, a DPP-4 inhibitor for the treatment of patients with type 2 diabetes: a review of recent clinical trials Curr Med Res Opin 2008 24 489 496 10.1185/030079908X261069 18182122

[B28] Alba M Ahren B Inzucchi SE Guan Y Mallick M Xu L Initial combination therapy with sitagliptin and pioglitazone: complementary effects on postprandial glucose and islet cell function (abstract) IDF Abstract Book 2009 P-1740 584

[B29] Yoon KH Shockey GR Teng R Golm GT Thakkar PR Meehan AG Sitagliptin and pioglitazone initial combination therapy in patients with type 2 diabetes provides substantial and durable incremental improvement in glycemic control over one year compared with initial treatment with pioglitazone monotherapy (abstract) IDF Abstract Book 2009 P-1153 391

[B30] Lovshin JA Drucker DJ Incretin-based therapies for type 2 diabetes mellitus Nat Rev Endocrinol 2009 5 262 269 10.1038/nrendo.2009.48 19444259

[B31] Vella A Bock G Giesler PD Burton DB Serra DB Saylan ML Effects of dipeptidyl peptidase-4 inhibition on gastrointestinal function, meal appearance, and glucose metabolism in type 2 diabetes Diabetes 2007 56 1475 1480 10.2337/db07-0136 17303799

[B32] Stevens JE Maddox AF Wolthers T Deacon C Nauck M Rayner CK Sitagliptin has no effect on gastric emptying in healthy humans (abstract) Australian Diabetes Society 2009 abstract # 95.

[B33] Hellstrom PM GLP-1: broadening the incretin concept to involve gut motility Regul Pept 2009 156 9 12 10.1016/j.regpep.2009.04.004 19362109

[B34] Richter B Bandeira-Echtler E Bergerhoff K Lerch CL Dipeptidyl peptidase-4 (DPP-4) inhibitors for type 2 diabetes mellitus Cochrane Database Syst Rev 2008 CD006739 18425967 10.1002/14651858.CD006739.pub2 PMC8985075

[B35] Kimmel D Cusick T Mu J Pennypacker B Shen XL Li ZH Thiazolidinediones, but not sitagliptin, exacerbate ovariectomy-induced bone loss in rats (abstract) Diabetes 2009 58 Suppl 1 A374

[B36] Haffner SM Lehto S Ronnemaa T Pyorala K Laakso M Mortality from coronary heart disease in subjects with type 2 diabetes and in nondiabetic subjects with and without prior myocardial infarction N Engl J Med 1998 339 229 234 10.1056/NEJM199807233390404 9673301

[B37] The University Group Diabetes Program A study of the effects of hypoglycemic agents on vascular complications in patients with adult-onset diabetes. Mortality results Diabetes 1970 19 Suppl 1 789 830 4926376

[B38] Nissen SE Wolski K Effect of rosiglitazone on the risk of myocardial infarction and death from cardiovascular causes N Engl J Med 2007 356 2457 2471 10.1056/NEJMoa072761 17517853

[B39] Bethel MA Green J Califf RM Holman RR Rationale and design of the Trial Evaluating Cardiovascular Outcomes with Sitagliptin (TECOS) (abstract) Diabetologia 2009 52 Suppl 1 S480

[B40] Giovannucci E Michaud D The role of obesity and related metabolic disturbances in cancers of the colon, prostate, and pancreas Gastroenterology 2007 132 2208 2225 10.1053/j.gastro.2007.03.050 17498513

[B41] Okolo S Incidence, aetiology and epidemiology of uterine fibroids Best Pract Res Clin Obstet Gynaecol 2008 22 571 588 10.1016/j.bpobgyn.2008.04.002 18534913

[B42] Pandya KA Radke F Benign skin lesions: lipomas, epidermal inclusion cysts, muscle and nerve biopsies Surg Clin North Am 2009 89 677 687 10.1016/j.suc.2009.03.002 19465204

[B43] European Medicines Agency (EMEA): Galvus (vildagliptin) - European Public Assessment Report (EPAR) - Scientific Discussion 2007 http://www.emea.europa.eu/humandocs/PDFs/EPAR/galvus/H-771-en6.pdf (last accessed February 9, 2010).

[B44] European Medicines Agency (EMEA): Onglyza (saxagliptin) - European Public Assessment Report (EPAR) - CHMP Assessment Report 2009 http://www.emea.europa.eu/humandocs/PDFs/EPAR/onglyza/H-1039-en6.pdf (last accessed February 9, 2010).

[B45] European Medicines Agency (EMEA): Januvia (sitagliptin) - European Public Assessment Report (EPAR) - Scientific Discussion 2007 http://www.emea.europa.eu/humandocs/PDFs/EPAR/januvia/H-722-en6.pdf (last accessed February 9, 2010).

[B46] Byrd JB Shreevatsa A Putlur P Foretia D McAlexander L Sinha T Dipeptidyl peptidase IV deficiency increases susceptibility to angiotensin-converting enzyme inhibitor-induced peritracheal edema J Allergy Clin Immunol 2007 120 403 408 10.1016/j.jaci.2007.04.012 17531305

[B47] Byrd JB Touzin K Sile S Gainer JV Yu C Nadeau J Dipeptidyl peptidase IV in angiotensin-converting enzyme inhibitor associated angioedema Hypertension 2008 51 141 147 10.1161/HYPERTENSIONAHA.107.096552 18025295 PMC2749928

[B48] Rosenstock J Brazg R Andryuk PJ Lu K Stein P Efficacy and safety of the dipeptidyl peptidase-4 inhibitor sitagliptin added to ongoing pioglitazone therapy in patients with type 2 diabetes: a 24-week, multicenter, randomized, double-blind, placebo-controlled, parallel-group study Clin Ther 2006 28 1556 1568 10.1016/j.clinthera.2006.10.007 17157112

[B49] Charbonnel B Karasik A Liu J Wu M Meininger G Efficacy and safety of the dipeptidyl peptidase-4 inhibitor sitagliptin added to ongoing metformin therapy in patients with type 2 diabetes inadequately controlled with metformin alone Diabetes Care 2006 29 2638 2643 10.2337/dc06-0706 17130197

[B50] Aschner P Kipnes MS Lunceford JK Sanchez M Mickel C Williams-Herman DE Effect of the dipeptidyl peptidase-4 inhibitor sitagliptin as monotherapy on glycemic control in patients with type 2 diabetes Diabetes Care 2006 29 2632 2637 10.2337/dc06-0703 17130196

[B51] Raz I Hanefeld M Xu L Caria C Williams-Herman D Khatami H Efficacy and safety of the dipeptidyl peptidase-4 inhibitor sitagliptin as monotherapy in patients with type 2 diabetes mellitus Diabetologia 2006 49 2564 2571 10.1007/s00125-006-0416-z 17001471

[B52] Williams-Herman D Johnson J Teng R Luo E Davies MJ Kaufman KD Efficacy and safety of initial combination therapy with sitagliptin and metformin in patients with type 2 diabetes: a 54-week study Curr Med Res Opin 2009 25 569 583 10.1185/03007990802705679 19232032

[B53] Williams-Herman D Johnson J Teng R Golm G Kaufman KD Goldstein BJ Efficacy and safety of sitagliptin and metformin as initial combination therapy and as monotherapy over 2 years in patients with type 2 diabetes Diabetes Obes Metab 2010 12 442 451 10.1111/j.1463-1326.2010.01204.x 20415693

[B54] Mohan V Yang W Son HY Xu L Noble L Langdon RB Efficacy and safety of sitagliptin in the treatment of patients with type 2 diabetes in China, India, and Korea Diabetes Res Clin Pract 2009 83 106 116 10.1016/j.diabres.2008.10.009 19097665

[B55] Barzilai N Mahoney EM Guo H Golm GT Williams-Herman D Kaufman KD Sitagliptin monotherapy is an effective and well-tolerated treatment for type 2 diabetes in elderly patients (abstract) 61st Annual Scientific Meeting of the Gerontological Society of America 2008

[B56] Aschner P Katzeff H Guo H Sunga S Williams-Herman D Kaufman KD Efficacy and safety of monotherapy of sitagliptin compared with metformin in patients with type 2 diabetes Diabetes Obes Metab 2010 12 252 261 10.1111/j.1463-1326.2009.01187.x 20070351

[B57] Dobs A Goldstein BJ Wieczorek L Golm G Davies MJ Williams-Herman D Triple combination therapy with sitagliptin, metformin, and rosiglitazone improves glycemic control in patients with type 2 diabetes (abstract) Diabetes 2008 57 Suppl 1 A595 A596

[B58] Raz I Chen Y Wu M Hussain S Kaufman KD Amatruda JM Efficacy and safety of sitagliptin added to ongoing metformin therapy in patients with type 2 diabetes Curr Med Res Opin 2008 24 537 550 10.1185/030079908X260925 18194595

[B59] Scott R Loeys T Davies MJ Engel SS Efficacy and safety of sitagliptin when added to ongoing metformin therapy in patients with type 2 diabetes Diabetes Obes Metab 2008 10 959 969 10.1111/j.1463-1326.2007.00839.x 18201203

