# Supplementary material for: Safety and tolerability of sitagliptin in clinical studies: a pooled analysis of data from 10,246 patients with type 2 diabetes
Source: BMC Endocr Disord. 2010 Apr 22;10:7. doi: 10.1186/1472-6823-10-7 (PMC3161395; doi:10.1186/1472-6823-10-7)
Supplement: Additional file 1 — Supplemental Table S1. Non-malignant adverse events in the Neoplasm SOC. The table lists the types and incidence rates of the non-malignant adverse events. [file 1472-6823-10-7-S1.PDF]

Additional file 1 (Williams-Herman et al.)

Supplemental Table S1. Non-malignant adverse events in the Neoplasm SOC

Adverse Event	n / patient-years of exposure (Incidence Rate per 100 Patient-years[†])		
	Sitagliptin 100 mg	Non-exposed	Difference between Sitagliptin and Non-exposed (95% CI)*
Any non-malignant adverse event in the Neoplasm SOC	62/4664 (1.3)	28/3926 (0.7)	0.6 (0.2, 1.1)
Acrochordon	1/4708 (0.0)	4/3943 (0.1)	-0.1 (-0.2, 0.0)
Adrenal adenoma	1/4709 (0.0)	0/3943 (0.0)	0.0**
Angiomyolipoma	1/4709 (0.0)	0/3943 (0.0)	0.0**
Benign breast neoplasm	1/4708 (0.0)	1/3943 (0.0)	-0.0**
Benign salivary gland neoplasm	1/4709 (0.0)	0/3943 (0.0)	0.0**
Bladder neoplasm	0/4709 (0.0)	1/3943 (0.0)	-0.0**
Carcinoid tumor of the gastrointestinal tract	1/4709 (0.0)	0/3943 (0.0)	0.0**
Carcinoid tumor of the small bowel	0/4709 (0.0)	1/3943 (0.0)	-0.0**
Cholesteatoma	1/4708 (0.0)	0/3943 (0.0)	0.0**
Colon adenoma	4/4705 (0.1)	0/3943 (0.0)	0.1 (-0.0, 0.2)
Fibroma	1/4708 (0.0)	0/3943 (0.0)	0.0**
Gastrointestinal tract adenoma	0/4709 (0.0)	1/3942 (0.0)	-0.0**
Glomus tumor	1/4708 (0.0)	1/3942 (0.0)	-0.0**
Hemangioma	1/4709 (0.0)	0/3943 (0.0)	0.0**
Hemangioma of liver	1/4708 (0.0)	0/3943 (0.0)	0.0**
Keratoacanthoma	1/4708 (0.0)	0/3943 (0.0)	0.0**
Leiomyoma/Uterine leiomyoma[‡]	11/2180 (0.5)	3/1725 (0.2)	0.4 (-0.0, 0.9)
Lipoma	8/4703 (0.2)	2/3942 (0.1)	0.1 (-0.0, 0.3)
Lung neoplasm	2/4708 (0.0)	1/3943 (0.0)	0.0**
Melanocytic nevus	5/4704 (0.1)	2/3941 (0.1)	0.1 (-0.1, 0.2)
Morton's neuroma	1/4708 (0.0)	0/3943 (0.0)	0.0**
Neoplasm skin	1/4709 (0.0)	1/3943 (0.0)	-0.0**
Neurilemmoma	1/4708 (0.0)	0/3943 (0.0)	0.0**
Ocular neoplasm	1/4709 (0.0)	0/3943 (0.0)	0.0**
Ovarian adenoma	1/4708 (0.0)	1/3943 (0.0)	-0.0**
Pancreatic neoplasm	0/4709 (0.0)	1/3943 (0.0)	-0.0**
Papilloma	1/4707 (0.0)	0/3943 (0.0)	0.0**
Prolactinoma	1/4708 (0.0)	0/3943 (0.0)	0.0**
Pyogenic granuloma	1/4708 (0.0)	0/3943 (0.0)	0.0**
Renal adenoma	1/4708 (0.0)	0/3943 (0.0)	0.0**
Retroperitoneal neoplasm	1/4709 (0.0)	0/3943 (0.0)	0.0**
Seborrheic keratosis	2/4707 (0.0)	4/3938 (0.1)	-0.1 (-0.2, 0.1)
Skin papilloma	5/4705 (0.1)	1/3943 (0.0)	0.1 (-0.1, 0.2)
Thyroid adenoma	1/4709 (0.0)	1/3942 (0.0)	-0.0**
Thyroid neoplasm	2/4706 (0.0)	2/3942 (0.0)	-0.0**

CI = confidence interval;

n = number of patients with ≥1 occurrence of the endpoint; CI = confidence interval;
† 100 * (number of patients with ≥1 event / person years of follow-up time).
* Between-group difference and 95% CI based on stratified analysis. Positive differences indicate that the incidence rate for the sitagliptin group is higher than the incidence rate for the non-exposed group. "0.0" and "-0.0" represent rounding for values that are slightly greater and slightly less than zero, respectively.
** 95% CIs were not computed for events that occurred in fewer than 4 patients in both groups, because the CIs would necessarily have included 0.
‡ The terms leiomyoma and uterine leiomyoma were combined because the only adverse event reported as leiomyoma was a case of uterine leiomyoma. Data are adjusted for gender-specific exposure.
